# Deterministic Approach to Achieve Full-Polarization Cloak

**DOI:** 10.34133/2021/6382172

**Published:** 2021-03-01

**Authors:** He-Xiu Xu, Yanzhao Wang, Chaohui Wang, Mingzhao Wang, Shaojie Wang, Fei Ding, Yongjun Huang, Xiaokuan Zhang, Haiwen Liu, Xiaohui Ling, Wei Huang

**Affiliations:** ^1^Air and Missile Defense College, Air Force Engineering University, Xi'an 710051, China; ^2^Institute of Flexible Electronics, Northwestern Polytechnical University, Xi'an 710072, China; ^3^College of Physics and Electronic Engineering, Hengyang Normal University, Hengyang 421002, China; ^4^SDU Nano Optics, University of Southern Denmark, Campusvej 55, DK-5230 Odense, Denmark; ^5^School of Information and Communication Engineering, University of Electronic Science and Technology of China, Chengdu 611731, China; ^6^School of Electronic and Information Engineering, Xi'an Jiaotong University, Xi'an 710049, China

## Abstract

Achieving full-polarization (*σ*) invisibility on an arbitrary three-dimensional (3D) platform is a long-held knotty issue yet extremely promising in real-world stealth applications. However, state-of-the-art invisibility cloaks typically work under a specific polarization because the anisotropy and orientation-selective resonant nature of artificial materials made the *σ*-immune operation elusive and terribly challenging. Here, we report a deterministic approach to engineer a metasurface skin cloak working under an arbitrary polarization state by theoretically synergizing two cloaking phase patterns required, respectively, at spin-up (*σ*+) and spin-down (*σ*−) states. Therein, the wavefront of any light impinging on the cloak can be well preserved since it is a superposition of *σ*+ and *σ*− wave. To demonstrate the effectiveness and applicability, several proof-of-concept metasurface cloaks are designed to wrap over a 3D triangle platform at microwave frequency. Results show that our cloaks are essentially capable of restoring the amplitude and phase of reflected beams as if light was incident on a flat mirror or an arbitrarily predesigned shape under full polarization states with a desirable bandwidth of ~17.9%, conceiving or deceiving an arbitrary object placed inside. Our approach, deterministic and robust in terms of accurate theoretical design, reconciles the milestone dilemma in stealth discipline and opens up an avenue for the extreme capability of ultrathin 3D cloaking of an arbitrary shape, paving up the road for real-world applications.

## 1. Introduction

Invisibility has been a long-standing dream for humanity for centuries until the proposal of transformation optics (TO) [1] and the advent of metamaterials [2]. Through these two strong mathematical and physical tools, perfect invisibility with light/electromagnetic (EM) wave flowing around a hidden object can be conceivable by precisely tailoring constitutive parameters of bulk material compositions with both anisotropy and inhomogeneity [3–6]. Although TO-meta grouped strategy is essentially a fascinating avenue toward invisibility, the bulky volume and singular constitutive parameters make it an inconvenient way for realization. Such an issue was later slightly mitigated by a transmission-line cloak in terms of energy coupled network [7] and plasmonic mantle cloak based on scattering cancellation technique in which the scattering wave radiated by the object to be cloaked was cancelled out by the antiphase response of thin metamaterial patterns [8–10]. Nevertheless, the size of the concealed objects was exceedingly limited in the former case, while in the latter case, the cloaking performance in terms of radar cross-section reduction preserved only in a narrow bandwidth, still hindering their practical applications in stealth discipline.

Fortunately, the invention of metasurfaces affords us a great degree of freedom (DoF) in controlling the local amplitude, phase, and polarization of a scattering EM wave, leading to many fascinating applications [11–20]. The significant advances in metasurfaces also render a great success in carpet cloak [21–28]. Therein, by wrapping over the object with an elaborately designed skin metasurface cloak, the phase and amplitude of the scattering wave can be restored to mimic a flat mirror as if the object does not exist. Recently, a tunable [29] and an intelligent cloak [30] was developed, which advances a grand step toward this realm. Nevertheless, achieving a deterministic full-polarization invisible cloak is still formidably challenging and remains a science fantasy. This is because any artificial material reacts to a special EM response only at a specific polarization (*σ*), rendering it detectable at other polarizations. To reconcile this dilemma, a three-dimensional (3D) pyramid cloak was reported by a cautious design of square ring meta-atoms with structural rotation symmetry [23], which indeed provides a desirable polarization-insensitive cloaking performance under linearly polarized (LP) wave with arbitrary polarization angle because any LP wave is a superposition of the transverse electric (TE) and transverse magnetic (TM) wave. However, it is impossible to preserve its polarization under circularly polarized (CP) wave detection, and thus, the cloaking effect would be much diminished since the ground-backed isotropic reflection scheme would reverse the handedness of the excited CP wave. Moreover, the above method is structure-dependent rather than a deterministic route, and the symmetry requirement poses a large limitation to anisotropic design with large DoF [31, 32]. To date, there is still no efficient methodology reported for this long-held pivotal and knotty issue.

**Figure 1:**
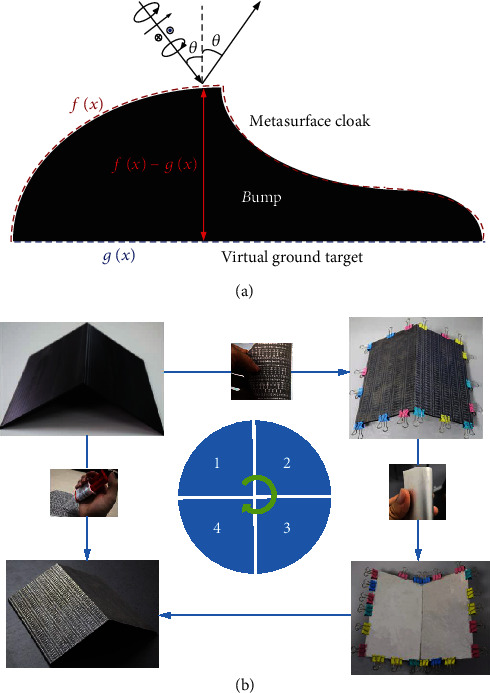
(a) Schematic and (b) fabrication process of our full-polarization metasurface cloak. The cloak sample is designed using an automatic computer-aided design (CAD) process and prepared based on a four-step dual-sided fabrication process by combining 3D-printing and flexible-printed circuit board (PCB) technique; see sample preparing and fabrication in Materials and Methods for details. The commonly available 0.1 mm-thick F4B board with *ε*_r_ = 2.65 and tan*δ* = 0.001 is utilized as the flexible thin substrate and backed ground. By taking the stability and rigidity into consideration, the 2.5 mm-thick polymer ABS-M30 with *ε*_r_ = 2.7 and tan*δ* = 0.005 is chosen as the 3D-printing material to preserve the perfect shape of the supporting platform, and thus the well-designed phase profiles.

Here, we reported counterintuitively an anisotropic avenue while a deterministic approach aimed at completely addressing this fundamental issue which should make a milestone on real-world stealth applications. As shown in Figure 1(a), the wave after impinging onto our metasurface cloak is always precisely scattered to the predicted mirror direction despite its initial polarization. Specifically, it works efficiently under the LP, CP, or even elliptical CP wave, manifesting unlimited polarization adaptability. The full-polarization operation is achieved by smartly imparting a set of phase patterns required simultaneously for left-handed/spin-up (LCP, *σ*+) and right-handed/spin-down (RCP, *σ*−) CP states. Therefore, our polarization-insensitive strategy is a completely emerging technique and is totally irrespective of four-fold (C4) rotation symmetry which was typically reported for conventional partial-polarization operation under two orthogonal LP states [23]. Moreover, as shown in Figure 1(b), the two-dimensional (2D) carpet cloak with invisibility along one incidence plane is realized by combining a flexible metasurface [33, 34] and a 3D printing technique [35] which is applicable with arbitrary complex structures and platforms. As such, our approach not only circumvents the *σ*-fragile cloaking dilemma but also considerably easy the fabrication, advancing a big step forward in real-world stealth applications.

## 2. Results

### 2.1. Principle and Design of the Full-Polarization Carpet Cloak

To not lose generality, we start from cloaking a black metallic bump with an arbitrary boundary described by the *f*(*x*) function (the dark red dashed shown in the schematic cloak of Figure 1(b)). By wrapping an elaborately engineered metasurface cloak on the bump, the scattering wavefront can be engineered the same as that of an arbitrary virtual target modelled by the *g*(*x*) function marked with the blue dashed line. The metasurface is a carpet cloak provided that *g*(*x*) is a constant function representing a horizontal ground. Otherwise, it is an illusion cloak which mimics a more complicated fake target. In both cases, the required compensated phase *δ* for a cloak targeted at specific frequency *f*_0_ and polarization *σ* can be theoretically calculated as [22]

(1) $$\begin{array}{c} \delta =\pi -2k_{0}\left[f\left(x\right)-g\left(x\right)\right]\cos\left(\theta \right). \end{array}$$

**Figure 2:**
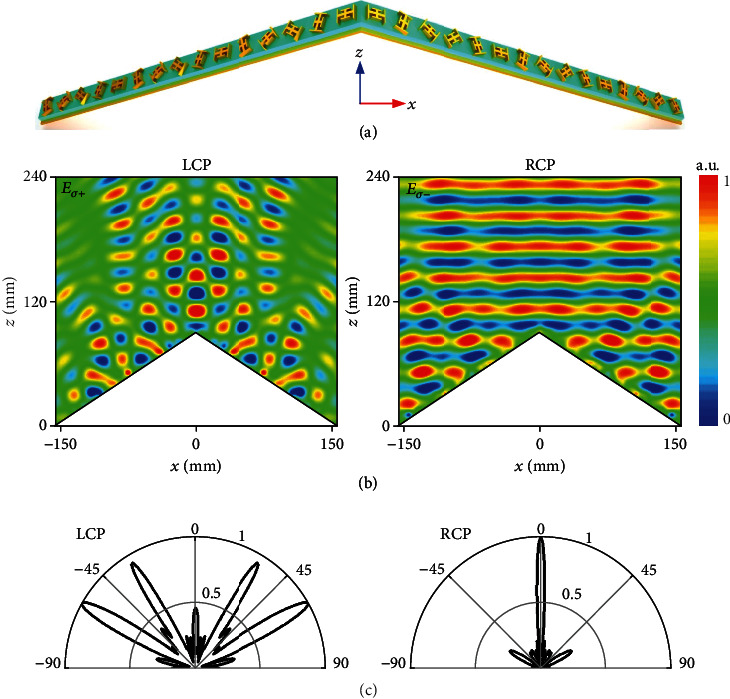
Illustration of the *σ*-sensitive cloaking performance using conventional metasurface approach. (a) Layout of the triangle cloak composed of 1 × 30 Jerusalem-cross meta-atoms based on PB phase. The triangle bump is with a tilted angle of *ψ* = 30° and a cross-section of *L* × *H* = 312 mm × 90 mm. There are a total of 15 meta-atoms along each slope of the triangle cloak. (b) FDTD-calculated copolarized NF *E*_*σ*+_ and *E*_*σ*−_ distributions and (c) FF *E*-field scattering patterns at 10.5 GHz when the cloaked bump is illuminated by a normally incident *σ*+ (left panel) and *σ*− (right panel) wave. Here, only the reflected field is plotted in NF patterns for clarity by subtracting the total field from the incident one. All NF and FF results are normalized to their maximum.

Here, *k*_0_ = 2*π*/*λ*_0_ is a free-space wavevector, and *θ* is the wave incidence angle. Therefore, the key for perfect cloaking performance is to engineer a precise phase profile on the skin metasurface sheet. However, the phase discontinuity of a meta-atom is commonly associated with its resonant feature in response to a specific *σ* wave. A slight change of *σ* will induce large or complete phase distortions which are doomed to deteriorate the eventual performance. To understand such a *σ*-sensitive issue in the conventional cloaking approach and lay a basis for our design, we first design a real metasurface cloak wrapping over a triangle metallic bump (Figure 2(a)) and analyze its invisible properties. The phase compensation is realized based on the geometric phase (PB phase) by rotating the Jerusalem cross meta-atom (see Supplementary materials for details). The metasurface cloak is targeted at 10.5 GHz under*σ*− wave and is normally triggered by *σ*+ and *σ*− waves, respectively. To characterize its invisibility property under different spin states, we numerically calculated the near-field (NF) and far-field (FF) results in FDTD simulation package CST Microwave Studio (see numerical characterizations in Materials and Methods). As shown in Figures 2(b) and 2(c), the plane wavefront with a narrow specular beam is expected under *σ*− wave excitation, indicating that the scattering fields are reconstructed with the same phase and amplitude as if the light were incident on a flat ground. However, the fields are completely distorted with four beams scattered into four directions in free space under *σ*+ wave illumination, which is totally different from those observed under the *σ*− wave case, convincing us an impressive *σ*-sensitive cloaking property.

The strategy we conceived to break this fundamental *σ*-sensitive issue is inspired from a hybrid approach, where complete decoupling of phases and functions is achieved for two orthogonal spin states in a copolarization system by combining both the geometric and dynamic phases [36, 37]. In fact, such phase decoupling between two spin states can be also implemented in a cross-LP system. If we can impart simultaneously two independent phase patterns (*δ*_*σ*+_ and *δ*_*σ*−_) to the metasurface under LCP and RCP state, respectively, then the cloak is expected to operate at any *σ* state with arbitrary polarization angle. This is quite physical because any incident EM wave can be decomposed into a superposition of *σ*+ and *σ*− waves. This property implies that the resulting cloak is polarization insensitive and is capable of rendering invisibility under any incident polarization. To verify our proposal, we first generalize the criterion of phase decoupling between two orthogonal spin states for both co-LP and cross-LP system (see Materials and Methods for the theoretical approach). Then, we devise an anisotropic cross-LP scheme to realize the aforementioned spin-decoupled phase patterns.

**Figure 3:**
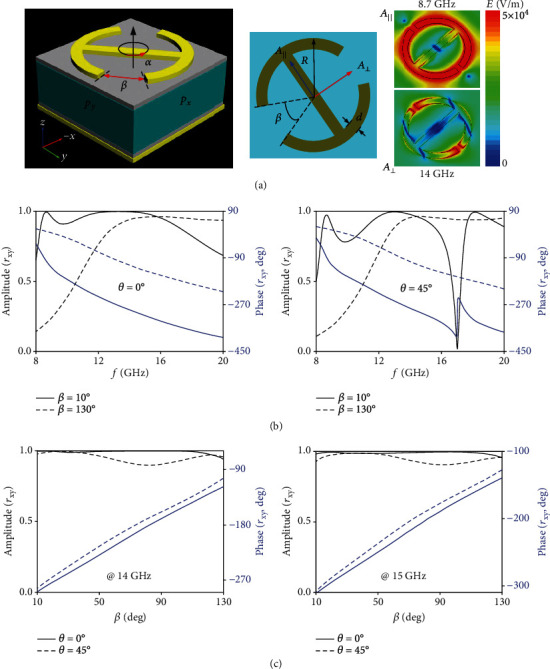
Design of the basic building block for our full-polarization carpet cloak. (a) Layout of the meta-atom with electric-field distributions corresponding to dual modes at 8.7 and 14 GHz shown in the inset. (b) FDTD-calculated cross-LP reflection amplitude and phase spectrum for meta-atoms of *β* = 10° and *β* = 130° under incidence angle of *θ* = 0° (left panel) and *θ* = 45° (right panel). (c) FDTD-calculated cross-LP reflection amplitude and phase versus *β* under *θ* = 0° and 45° at the frequency of 14 (left panel) and 15 GHz (right panel). The detailed geometric parameters are *p*_x_ = *p*_y_ = 5.5 mm, *d* = 0.4 mm, and *R* = 2.2 mm. The utilized 3D printing material is ABS-M30 with a dielectric constant *ε*_*r*_ = 2.7, loss tangent tan*δ* = 0.005, and thickness *h* = 2.5 mm while the top and bottom flexible dielectric layer is a F4B board with *ε*_*r*_ = 2.65, tan*δ* = 0.001, and *h* = 0.1 mm.

The basic building block utilized for our full-polarization carpet cloak is a metal-insulator-metal reflection meta-atom composed of a flexible thin substrate and a 3D printing polymer material ABS-M30 sandwiched by top quasi-I-shaped metallic pattern and bottom flat ground, as portrayed in Figure 3(a). The top metallic pattern [38], etched on the flexible board, is a composite of the central bar and end-loaded split-ring resonators (SRRs) characterized by an open-angle *β* and an orientation angle of *α* = 45° with respect to the *x*-axis. The commonly available 0.1 mm-thick F4B board is utilized as the flexible thin substrate and backed ground, which is finally attached to the ABS-M30 polymer by adhesives. Such a fabrication process avoids metallizing the 3D-printed substrates through thin film sputtering [27]. By taking the stability and rigidity into consideration, the thickness of ABS-M30 is chosen as *h* = 2.5 mm to preserve the perfect shapes of supporting materials, and thus the well-designed phase profiles.

Similar to V-shaped meta-atoms [11], two modes (*A*_//_ and *A*_⊥_) can be simultaneously excited parallel and perpendicular to the *I*-oriented axis under the *x*- or *y*-polarized LP wave. The cascading of these two interelement modes, identified from two cross-LP *r*_*xy*_ peaks, can be smartly employed to engineer a broadband high-efficiency cross-LP meta-atom. As shown in Figure 3(b) and Figure S1, the meta-atom with *α* = 45° and *β* = 10° exhibits a high cross-LP rate with *r*_*xy*_ more than 0.85 across 8.4~18.9 GHz under normal incidence, corresponding to a fractional bandwidth of 77%. The near-unity cross-LP reflection indicates an out-of-phase difference between the above two anisotropic modes which is the key to preserve the handedness of the triggered CP wave [39]. The mechanism lies in that anisotropy-induced CP conversion here will compensate the inherent ground triggered CP conversion. Moreover, by changing *β* from 10° to 130°, the continuous dynamic phase change of *φ*_*xy*_ with a maximum of 180° can be achieved in the above broad frequency range. To satisfy a full 2*π* phase cover, the additional 180° phase jump can be continued via changing *α* by 90° without altering *r*_*xy*_ significantly. Most importantly, the phase response is insensitive to the incidence angle *θ* as depicted in Figure 3(c), which exhibits a maximum phase tolerance of 15° and 20° at 14 and 15 GHz when *θ* alters from 0° to 45°. Such an angle-immune phase response is particularly beneficial for a cloak of arbitrary boundary, where the phase errors induced by different *θ* of spatially varied meta-atom can be minimized. Although a slight amplitude error is observed at *θ* = 45°, the reflection rate is still above 0.85 for all *β* which poses negligible effect on the preserved amplitude of metasurface cloak.

Given the established clear principle, generalized sophisticated theory, and the unique EM characteristic of the basic building block, it is ready to implement our full-polarization cloak by imparting theoretically required dual-phase patterns *δ*_*σ*+_ and *δ*_*σ*−_ onto our metasurface. Here, we choose a triangle platform to design our metasurface skin cloak for demonstration. It is a stacked composite of ABS-M30 and a thin F4B metallic ground and is characterized by the length *P*, cross-section tilt angle *ψ*, and width *L*. Given the geometric parameters of triangle bump, the theoretically required dual-phase patterns *δ*_*σ*+_ and *δ*_*σ*−_ can be immediately achieved according to Eq. (1). Then, the required dynamic phase *φ*_*xy*_ and geometric phase 2*α* can be readily synthesized by following the spin-decoupled theory established for the cross-LP system. Finally, the layout of our thin metasurface cloak composed of spatially varied meta-atoms can be mapped out according to the target *φ*_*xy*_ (*β*) and *α* distributions through a CAD process. For comprehensive verification, we designed several metasurface cloaks and characterized their invisibility property based on FDTD-calculated and experimentally measured NF and FF results under different excitation scenarios. Detailed information can be referred to the numerical characterizations, sample fabrication, and microwave experiments in Materials and Methods.

### 2.2. Full-Polarization Metasurface Illusion Device

**Figure 4:**
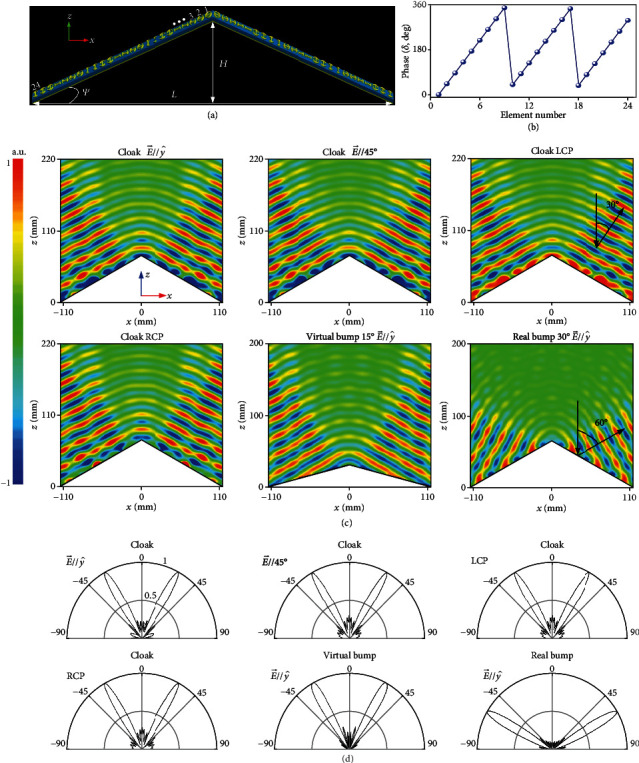
FDTD characterization of the metasurface illusion device at 14 GHz under a normal incidence of different polarization states. (a) Layout and (b) phase profile along the centered *x*-axis. Here, the phase distribution is given only for the left half of the cloak due to its symmetry for the residual half counterpart. The metasurface cloak is infinite along the *y* direction by imposing a periodic boundary. FDTD-calculated (c) the NF *E*_y_ distributions and (d) copolarized (the same polarization as the excitation) the FF *E*-field scattering patterns in *xz* plane for both cloaked bump and bare metallic bump of the same size under $\vec{E}//\hat{y}$ (*σ*_*y*_), $\vec{E}//45^{{}^{\circ}}$, LCP, and RCP. Here, the NF and FF results of the cloaked bump under $\vec{E}//\hat{x}$ (*σ*_*x*_) are almost the same as those under available *σ* states and are not given here for the brevity of contents, see Supplementary Figures S3a and S4. The real triangle bump to be cloaked is with a tilted angle of *ψ* = 30° and a cross-section of *L* × *H* = 228.6 mm × 68.5 mm. The virtual EM shape to be emulated is also a metallic triangle bump with a tilted angle of *ψ* = 15° and a cross-section of *L* × *H* = 228.6 mm × 34.2 mm. There are a total of 24 meta-atoms along each slope of the illusion device.

We first design a full-polarization illusion device [40, 41] which can be considered as a special case of a cloak. Such an illusion concept finds unprecedented applications in deception jamming of an electronic warfare and coincides well with that of camouflage [42] in terms of mimicking the scattering signature of other arbitrarily predefined objects. Although a broadband camouflage device is realized in wide-angle operation through optical surface transformation [42], the cloak works still under a specific polarization detection and the lateral displacement is unavoidable due to the bulk volume. Instead, our illusion device is with skin thickness and is polarization insensitive. It is targeted at 14 GHz on a real triangle platform characterized by *ψ* = 30° and a cross-section of *L* × *H* = 228.6 mm × 68.5 mm in the triple-side cross-section. The virtual EM shape to be emulated is also a triangle bump with an identical length of *L* = 228.6 mm but a different tilted angle of *ψ* = 15° (*H* = 34.2 mm).  Figure 4(a) shows the layout of our designed cloak wrapped over a triangle metallic bump according to the phase profile calculated in Figure 4(b). As is shown, our cloak is assembled by two sub-metasurfaces composed of specific meta-atoms with spatially varied *β* and alternatively changed *α* = 0° and 90°. Indeed, the modified *α* from *α* = 45° to 0° and 90° according to the spin-decoupled theory plus the out-of-phase difference between two anisotropic modes of each meta-atom plays a key role in preserving the polarization of both LP and CP wave. The physics can be understood that the EM response of the above specific meta-atoms with 0° and 90° orientation is the same under *σ*_+_ and *σ*_−_ wave in terms of equal *e*^*i*2*α*^ and *e*^−*i*2*α*^. Such feature guarantees simultaneously preserved phase and amplitude for all polarizations which distinguishes our design from any existing metasurface cloak [22–30]. In FDTD characterizations, the bare bump and cloaked bump are normally illuminated by *y*- and 45°-polarized LP wave as well as *σ*+ and *σ*− plane wave incidents in the *xz* plane. As depicted in Figures 4(c) and 4(d), both the FDTD-calculated NF and FF results reveal that our illusion device manifests almost the same scattering wavefronts and beams as those of the virtual triangle bump under *y*/45°-aligned LP wave and *σ*+/*σ*− CP wave, indicating an elegant full-polarization illusion behavior. The scattering beam is symmetrically directed to ±30°, which is an exact mirror reflection angle 2*ψ* of the triangle virtual bump while it is altered to ±60° when our metasurface cloak is removed. The artificially engineered scattering signature by our cloak enables the real physical shape to be perceived to a virtual shape designed at will, indicating unprecedented applications to camouflage and bait to a critically important military target in defense security. It should be strengthened that other complicated scattering signatures of EM shape can be envisioned under full polarizations provided that the compensated phase pattern induced between the real and virtual objects can be reasonably evaluated, which is out the scope of this work. Moreover, the metasurface illusion device exhibits an elegant operation bandwidth of ~2 GHz within 13~15 GHz, corresponding to a fractional bandwidth of 14.3% (see Supplementary Figures S3 and S4 for more FDTD NF and FF results at other frequencies).

### 2.3. Full-Polarization Metasurface Carpet Cloak

**Figure 5:**
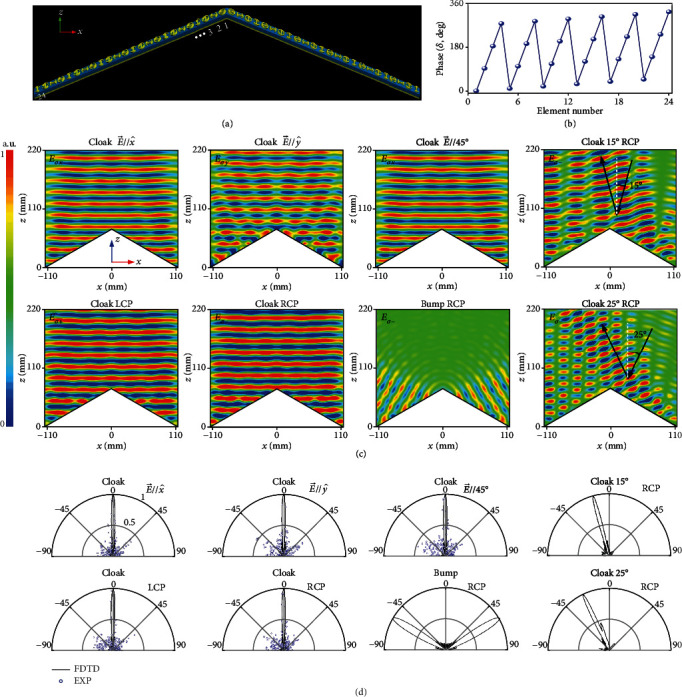
FDTD characterization of the metasurface carpet cloak at 14 GHz under normal, 15°, and 25° oblique incidence of different polarization states. (a) Layout and (b) phase profile along the centered *x*-axis. Here, the phase profile is given only for half of the cloak since it is symmetric about the *x*-axis for the left half counterpart. (c) FDTD-calculated copolarized NF contour maps and (d) comparison of copolarized FF *E*-field scattering patterns in the *xz* plane between the FDTD simulations and experiments for both cloaked and bare bump under $\vec{E}//\hat{x}$ (*σ*_*x*_), $\vec{E}//\hat{y}$ (*σ*_*y*_), $\vec{E}//45^{{}^{\circ}}$, LCP, and RCP wave. The triangle bump is with a tilt angle of *ψ* = 30° and a cross-section of *L* × *H* = 228.6 mm × 68.5 mm. There are a total of 24 meta-atoms along each slope of the carpet cloak.

To further verify the robustness of our approach, we design a carpet cloak that is capable of mimicking the scattering feature of a metallic ground by utilizing the previous triangle platform.  Figure 5(a) portrays the layout of our designed full-polarization cloak wrapping over a triangle metallic bump according to the phase profile calculated in Figure 5(b). Again, our metasurface cloak comprises spatially varied meta-atoms with distinct dimensions and orientations, which distinguishes our work from any available report. As is depicted in Figures 5(c) and 5(d), flattened reflective wavefronts with near-uniform intensity and highly directive single-mode specular scattering are clearly inspected under *x*-, *y*- and 45°-polarized LP wave and *σ*+/*σ*− CP wave, indicating that distorted reflection fields after impinging on the cloak are well restored similar to those from a flat metallic plate. Therein, any object placed inside the cloak can be completely invisible from the background under detection of all polarized wave/light. By contrast, two tilted wavefronts directed toward two strongly scattered pencil beams are clearly observed if no metasurface cloak is available, rending the bump exposed to be completely detectable. Moreover, our cloak enables to work without degenerated performance even under oblique incidence up to *θ* = 25°, where mirror reflections are clearly observed. The insensitive phase response of our meta-atom gives rise to the off-normal operation. Substantial phase errors will be induced for larger *θ* since the required *δ* is *θ*-dependent, which needs precise design to compensate.

**Figure 6:**
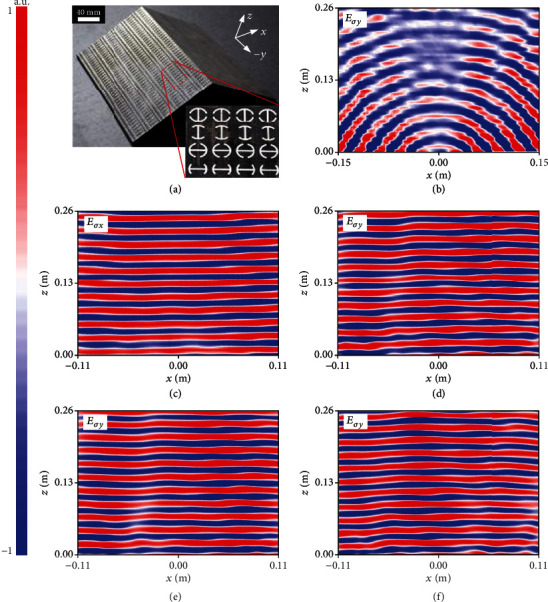
Experimental characterization of the metasurface carpet cloak at 14.5 GHz under normal incidence of different polarization states. (a) Photograph of the fabricated sample with the magnified picture shown in the inset. Here, a total of 40 pixels is periodically repeated along y axes to eliminate the finite-size truncation effect, corresponding to an aperture of 220 mm. NF contour maps of the (b) bare metallic bump under $\vec{E}//\hat{y}$ and (c–f) the cloaked bump under (c) $\vec{E}//\hat{x}$, (d) $\vec{E}//\hat{y}$, (e) LCP, and (f) RCP state.

Figure 6(a) shows the photograph of the finally assembled metasurface skin cloak by our synergetic strategy of PCB and 3D-printing technique. A total of 40 pixels is periodically repeated along the *y*-axes to eliminate the finite-size truncation effect, corresponding to an aperture of 220 mm. Figures 6(b)–6(f) depict the experimentally measured NF *E*-field maps for both bare bump and cloaked bump at 14.5 GHz by scanning an area of 0.22 × 0.26 m^2^ in the *xz* plane. As is much appreciated, all NF results are in good consistency with the FDTD simulations under *σ*_*x*_, *σ*_*y*_, *σ*_+_, and *σ*_−_ plane wave illumination except that the center cloaking frequency has shifted slightly from 14 GHz to 14.5 GHz in measurements. From Figure 6(b), it is learned that the reflected beam has been distorted and split into various directions. In sharp contrast, different split beams rejoin and manifest almost flat wavefront with uniform intensity in all scenarios of *σ* when the bump is covered with our metasurface cloak (Figures 6(c)–6(f)), revealing that the distortion of the reflected phase and wavefront is reconstructed for all inspected polarizations. This is quite intriguing with respect to [22], where the object is perfectly hidden only for *x* polarization and is completely visible by switching *σ*. The well-recovered wavefront can be further evidenced from the measured highly directive single-mode FF specular scattering patterns shown in Figure 5(d), where reasonable agreement is observed between FDTD calculation and experimental data. The slightly larger sidelobe and wider beamwidth in the latter case are attributed to the nonideal plane wave excitation and insufficient directivity of the triggered horn. Again, our metasurface carpet cloak exhibits an elegant bandwidth of 2/2.5 GHz within 13~15/13~15.5 GHz (experiment/FDTD), corresponding to a fractional bandwidth of 14.3/17.9% (see Supplementary Figures S5–S7 for more NF and FF results at other frequencies). Such a level of bandwidth is very considerable relative to existing metasurface cloaks [22–30].

### 2.4. Full-Polarization Large-Angle Metasurface Cloak

**Figure 7:**
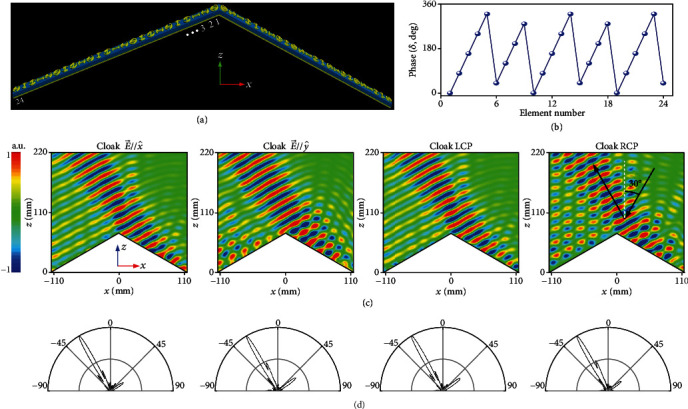
FDTD characterization of the large-angle metasurface carpet cloak at 14 GHz under an oblique incidence of *θ* = 30° in the *xz* plane at different polarization states of $\vec{E}//\hat{x}$, $\vec{E}//\hat{y}$, LCP, and RCP wave. (a) Layout and (b) phase profile along the centered *x*-axis. Here, the phase profile is given only for half of the cloak since it is symmetric about the *x*-axis for the left half counterpart. The triangle bump is with a tilted angle of *ψ* = 30° and a cross-section of *L* × *H* = 228.6 mm × 68.5 mm. There are a total of 24 meta-atoms along each slope of the metasurface cloak. (c) FDTD-calculated copolarized NF contour maps and (d) copolarized FF *E*-field scattering patterns in the *xz* plane under $\vec{E}//\hat{x}$ (*σ*_x_), $\vec{E}//\hat{y}$ (*σ*_*y*_), LCP, and RCP wave.

Finally, our full-polarization metasurface skin cloak can also be specially designed to work under large oblique incidence. Again, the 2D cloak in Figure 7(a) with spatially varied meta-atoms along *x* direction is implemented according to the phase profile shown in Figure 7(b). Figures 7(c) and 7(d) portray the FDTD-calculated NF and FF results of our cloak designed at *θ* = 30° under *x*-/*y*-polarized LP waves and *σ* + /*σ*− CP waves. As is shown, the outgoing flattened wavefront is tilted toward *θ* = −30° for all examined polarizations, indicating a mirror operation of the incident wave. Moreover, the FF scattered patterns precisely steered at an oblique angle of *θ* = −30° with considerably suppressed sidelobes, revealing a perfect mirror-reflection cloaking behavior. As discussed in Figure 3(c), large fluctuations of reflection amplitude would be induced for meta-atoms at oblique incidence. This is especially true for the nonuniform amplitudes of meta-atoms at two slopes induced by much-deviated incidence angle (*θ* = 90° and 0° for meta-atoms at left and right slopes) at oblique incidence. Such asymmetry nonuniform amplitudes of meta-atoms lead to sharply varied cloaking performance (deteriorative wavefront and large sidelobes) as frequency deviates from the center one, leading to a narrower operation bandwidth of 1 GHz (see Supplementary Figure S8).

## 3. Discussion

To sum up, we have proposed and verified numerically and experimentally a deterministic strategy for full-polarization invisible cloaking. Such a formidably challenging polarization-immune issue is theoretically addressed by imposing two sets of cloaking phases on two decoupled orthogonal spins. For verification, we have designed several sophisticated metasurface thin cloaks on a triangle platform by combining a 3D printing technique and a flexible PCB technique. Results reveal that our metasurface skin cloaks are capable of preserving the predesigned scattering signatures (both amplitude and phase) across an elegant bandwidth under fully polarized light detection. Moreover, the constraints raised by impedance mismatching and the lateral shift of the reflected beams in existing invisibility cloaks are also mitigated. Theoretically and practically, there is no cloaking shape and size limitation endowed by the fabrication. More importantly, our skin cloak possesses a superthin profile of ~*λ*/200, paving up the way for high-speed aerospace applications where light weight and aerodynamics are a major concern. Our strategy, deterministic and robust, completely addressed the long-held knotty issue of polarization-dependent cloaking and opened up an unprecedented avenue for the extreme capability of ultrathin cloaking of an arbitrary shape, advancing a giant step toward real-world stealth applications. Note that in the revision stage of this work, a spherical 3D cloak with a noneuclidean metasurface is reported independent of the azimuthal angle [43]. However, it still cannot work under CP wave detection, the same issue as discussed previously [23]. A 3D full-polarization metasurface cloak on a pyramid platform with invisibility along quasi-full azimuthal planes is in development based on our robust deterministic approach.

## 4. Materials and Methods

### 4.1. Numerical Characterizations

All numerical designs and FDTD characterizations are performed through numerical simulation package CST Microwave Studio. Specifically, in calculations of the reflection amplitudes/phases of the meta-atoms, especially in generating the reflection response database, we imposed periodic boundary conditions at its four bounds and placed a Floquet port 15 mm away from the meta-atom plane in the frequency-domain solver of the commercial software. In the NF and FF numerical characterizations of the *σ*-sensitive and full-polarization 2D triangle cloaks, the metasurfaces composed of 30 × 1 and 48 × 1 spatially varied meta-atoms along two slopes are utilized in time-domain solver with periodic boundary condition assigned to the *y* side to reduce the calculation volume, while four open boundaries set along the *x* and *z* axes.

### 4.2. Theoretical Approach for Full-Polarization Metasurface Skin Cloak

In the following, we generalize the theory to decouple phases at *σ*+ and *σ*− states for both co-LP and cross-LP system, aiming to afford the basic design criterion for full-polarization invisibility in reflection geometry. Suppose an arbitrary meta-atom rotating an angle of *α* with respect to its central axis under the Cartesian coordinate system, then the emerging linear reflection complex Jones matrix *R*(*α*) can be formulated as a function of its previous counterpart as *R*(*α*) = *S*^−1^(*α*) × *R* × *S*(*α*), where $S\left(\alpha \right)=\left(\begin{array}{cc} \cos\alpha & -\sin\alpha \\ \sin\alpha & \cos\alpha \end{array}\right)$. However, such a Jones matrix is derived as *R*^*c*^(*α*) = *Λ*^−1^•*R*(*α*)•*Λ* in circular polarization (CP) basis under *σ*+ and *σ*− state, where $\Lambda =\left(1/\sqrt{2}\right)\left(\begin{array}{cc} 1 & 1\\ -j & j \end{array}\right)$. The exotic feature of an arbitrary meta-atom is indicated by a general matrix $R=\left(\begin{array}{cc} r_{xx}e^{j\varphi_{xx}} & r_{xy}e^{j\varphi_{xy}}\\ r_{yx}e^{j\varphi_{yx}} & r_{yy}e^{j\varphi_{yy}} \end{array}\right)$, where *φ*/*r*_*xx*_, *φ*/*r*_*yx*_, *φ*/*r*_*xy*_, and *φ*/*r*_*yy*_ are phase and amplitude spectrum of four *x*/*y*-polarized reflection components under the excitation of *x*/*y* LP wave. These phases are corresponding to the dynamic phases associated with structure parameters. For a reciprocal system without complete rotational and mirror symmetry breaking discussed here, we have *r*_*yx*_ = *r*_*xy*_ and *φ*_*xy*_ = *φ*_*yx*_. Then, the reflection Jones matrix in CP basis can be formulated as

(2) $$\begin{array}{c} R^{c}\left(\alpha \right)=\left(\begin{array}{cc} r_{++}e^{j\delta_{++}} & r_{+-}e^{j\delta_{+-}}\\ r_{-+}e^{j\delta_{-+}} & r_{--}e^{j\delta_{--}} \end{array}\right)=\left(\begin{array}{cc} \frac{1}{2}\left(r_{xx}e^{j\varphi_{xx}}+r_{yy}e^{j\varphi_{yy}}\right) & \frac{1}{2}e^{-2\alpha j}\left(2{jr}_{yx}e^{j\varphi_{xy}}+r_{xx}e^{j\varphi_{xx}}-r_{yy}e^{j\varphi_{yy}}\right)\\ \frac{1}{2}e^{2\alpha j}\left(-2{jr}_{yx}e^{j\varphi_{xy}}+r_{xx}e^{j\varphi_{xx}}-r_{yy}e^{j\varphi_{yy}}\right) & \frac{1}{2}\left(r_{xx}e^{j\varphi_{xx}}+r_{yy}e^{j\varphi_{yy}}\right) \end{array}\right). \end{array}$$

Eq. (2) reveals that the involving of both geometric phase (*e*^−2*αj*^) and dynamic phase enables completely decoupled *δ*_*σ*+_ and *δ*_*σ*−_.

In a complete co-LP system with mirror symmetry along the *x* and *y* axis, there is no cross-polarization (*r*_*yx*_ ≈ *r*_*xy*_ ≈ 0), and we can easily engineer *r*_*xx*_ ≈ *r*_*yy*_ ≈ 1. Moreover, the term carrying geometric phase should be unity while the residual *r*_++_ and *r*_−−_ ought to approach zero in order to facilitate a high spin-conversion efficiency which is dependent on copolarization rate. Taking these aspects into consideration, we impose *φ*_*xx*_ − *φ*_*yy*_ ≈ *π* and further simplify Eq. (1) as $R^{c}\left(\alpha \right)=\left(\begin{array}{cc} 0 & e^{\varphi_{xx}-2\alpha j}\\ e^{\varphi_{xx}+2\alpha j} & 0 \end{array}\right)$. In this case, we deduce the required dynamic and geometric phase profiles to cloak objects under dual-spin states.

(3a) $$\begin{array}{c} \varphi_{xx}=\frac{1}{2}\left(\delta_{\sigma +}+\delta_{\sigma -}\right), \end{array}$$

(3b) $$\begin{array}{c} \varphi_{yy}=\frac{1}{2}\left(\delta_{\sigma +}+\delta_{\sigma -}\right)-\pi , \end{array}$$

(3c) $$\begin{array}{c} \alpha =\frac{1}{4}\left(\delta_{\sigma +}-\delta_{\sigma -}\right). \end{array}$$

On the contrary, for a complete cross-LP system without mirror symmetry, i.e., *r*_*xx*_ ≈ *r*_*yy*_ ≈ 0, we immediately obtain $R^{c}\left(\alpha \right)=\left(\begin{array}{cc} 0 & r_{xy}e^{j\left(\varphi_{xy}-2\alpha +\pi /2\right)}\\ r_{xy}e^{j\left(\varphi_{xy}+2\alpha -\pi /2\right)} & 0 \end{array}\right)$ and conclude that spin-decoupling efficiency is determined by the cross-LP rate. Then, the required LP phase patterns to achieve simultaneous invisibility at *σ*+ and *σ*− states are synthesized as

(4a) $$\begin{array}{c} \varphi_{xy}=\varphi_{yx}=\frac{1}{2}\left(\delta_{\sigma +}+\delta_{\sigma -}\right), \end{array}$$

(4b) $$\begin{array}{c} \alpha =\frac{1}{4}\left(\delta_{\sigma +}-\delta_{\sigma -}+\pi \right). \end{array}$$

### 4.3. Sample Fabrication and Microwave Experiments

The cloak sample is prepared based on a four-step dual-sided fabrication process by combining 3D-printing and PCB technique. The supporting triangle platform with specific tilted angles are prepared using 2.5 mm-thick 3D printing polymer material ABS-M30 (dielectric constant *ε*_r_ = 2.7 and loss tangent tan*δ* = 0.005) through 3D-printing technique, see Figure 1(b). The top and bottom metallic patterns and ground of our metasurface cloak were fabricated individually on two 0.1 mm-thick flexible F4B dielectric boards (*ε*_r_ = 2.65 and tan*δ* = 0.001) using the PCB technique. A CAD process is established which can automatically construct all metallic patterns through program codes in a commercial software based on the reflection response database and position database of each meta-atom. After all PCB boards and supporting platforms are ready, the next step is to align and attach each flexible board to two sides of the ABS-M30 platform to form an entirety through adhesives. Finally, they are shaped and reinforced by clamps for several hours. Such an assembling process avoids metallizing the 3D-printed substrates through thin film sputtering.

All FF and NF experiments are performed in a microwave anechoic chamber to avoid possible interference from the environment, see the experimental setup shown in Supplementary Figure S9. Two pairs of highly directive LP or CP antenna emitting Gaussian wave were utilized as receiver and transmitter. The double-ridged horn exhibiting a voltage standing wave ratio (VSWR) less than 2.5 within the frequency range 1–18 GHz is utilized as the LP antenna. By altering the orientation of the emitting antenna with respect to the fixed sample, an LP wave excitation can be readily realized with several representative polarization angles of 0°, 30°, 45°, and 90°. For CP wave excitation, the sample was illuminated by a horn with an axial ratio of less than 3.5 dB and a voltage-standing-wave ratio of less than 2.5 within 8~18 GHz.

In all NF contour maps, a 10/15 mm-long monopole, functioning as the receiver, was placed between the 1 m-distanced sample and horn and was connected to an AV3672B Agilent vector network analyzer to record the static EM signals. It was fixed to a 2D electronic step motor that can move automatically in a maximum area of 1.2 m × 1.2 m with a step resolution of 5 mm. To guarantee the pure scattering signature, the incident signal in free space was deducted from the total fields. In the FF scattering pattern measurements, the cloak sample and the receive horn to record signals were fixed on a large rigid foam which is capable of rotating freely along the foam's axial center. The transmitting horn was placed 6 m away to afford the desired excitations.

## Data Availability

The data used to support the findings of this study are available from the corresponding author upon request.

## Supplementary Materials

Supplementary Materials Figure S1 Topology and EM properties of the PB meta-atom utilized to construct the *σ*-sensitive metasurface cloak. Figure S2: FDTD-calculated cross-LP reflection amplitude (|*r*_*xy*_|) and phase (*φ*_*xy*_) of a basic meta-atom versus frequency and the parameter *β* under normal (*θ* = 0°) and oblique (*θ* = 45°) illumination. Figure S3: FDTD-calculated copolarized NF distributions in *xz* cross-section plane of the 2D metasurface illusion device at different frequencies of 13, 13.5, 14.5, and 15 GHz under normal incidence of $\vec{E}//\hat{x}$, $\vec{E}//\hat{y}$, $\vec{E}//45^{{}^{\circ}}$, LCP, and RCP wave. Figure S4: FDTD-calculated copolarized FF scattering patterns in *xz* cross-section planes of the 2D metasurface illusion device at different frequencies of 12.5~15 GHz in steps of 0.5 GHz under a normal incidence of $\vec{E}//\hat{x}$. Figure S5: FDTD-calculated copolarized NF distributions in the *xz* cross-section plane of the 2D metasurface carpet cloak at different frequencies of 13, 13.5, 14.5, and 15 GHz under the normal incidence of $\vec{E}//\hat{x}$, $\vec{E}//\hat{y}$, $\vec{E}//45^{{}^{\circ}}$, LCP, and RCP wave. Figure S6: Experimentally measured NF distributions in the *xz* cross-section plane of the (a) bare bump under $\vec{E}//\hat{y}$ and metasurface carpet cloak at different frequencies of 13, 15, and 15.5 GHz under normal incidence of (b) $\vec{E}//\hat{x}$, (c) $\vec{E}//\hat{y}$, (d) LCP, and (e) RCP wave. The *E*_y_ maps are plotted in $\vec{E}//\hat{y}$, LCP, and RCP cases while *E*_x_ maps are in the $\vec{E}//\hat{x}$ case. Figure S7: FDTD-calculated and experimentally measured copolarized FF scattering patterns in the *xz* cross-section planes of the metasurface carpet cloak at different frequencies of 12.5~16 GHz in steps of 0.5 GHz under normal incidence of $\vec{E}//\hat{y}$. Figure S8: FDTD-calculated copolarized NF contour maps (top panel) and copolarized FF scattering patterns (bottom panel) in the *xz* plane of the large-angle metasurface cloak at different frequencies of 13.5 (left panel) and 14.5 GHz (right panel) under oblique incidence (*θ* = 30°) of (a) $\vec{E}//\hat{x}$, (b) $\vec{E}//\hat{y}$, (c) LCP, and (d) RCP wave. Figure S9: Experimental setup for (a) NF and (b) FF measurements.
