# Supplementary material for: Deterministic Approach to Achieve Full-Polarization Cloak
Source: Research (Wash D C). 2021 Mar 1;2021:6382172. doi: 10.34133/2021/6382172 (PMC7945686; doi:10.34133/2021/6382172)
Supplement: Supplementary Materials — Figure S1 Topology and EM properties of the PB meta-atom utilized to construct the σ-sensitive metasurface cloak. Figure S2: FDTD-calculated cross-LP reflection amplitude (|rxy|) and phase (φxy) of a basic meta-atom versus frequency and the parameter β under normal (θ = 0°) and oblique (θ = 45°) illumination. Figure S3: FDTD-calculated copolarized NF distributions in xz cross-section plane of the 2D metasurface illusion device at different frequencies of 13, 13.5, 14.5, and 15 GHz under normal incidence of E→//x^, E→//y^, E→//45°, LCP, and RCP wave. Figure S4: FDTD-calculated copolarized FF scattering patterns in xz cross-section planes of the 2D metasurface illusion device at different frequencies of 12.5~15 GHz in steps of 0.5 GHz under a normal incidence of E→//x^. Figure S5: FDTD-calculated copolarized NF distributions in the xz cross-section plane of the 2D metasurface carpet cloak at different frequencies of 13, 13.5, 14.5, and 15 GHz under the normal incidence of E→//x^, E→//y^, E→//45°, LCP, and RCP wave. Figure S6: Experimentally measured NF distributions in the xz cross-section plane of the (a) bare bump under E→//y^ and metasurface carpet cloak at different frequencies of 13, 15, and 15.5 GHz under normal incidence of (b) E→//x^, (c) E→//y^, (d) LCP, and (e) RCP wave. The Ey maps are plotted in E→//y^, LCP, and RCP cases while Ex maps are in the E→//x^ case. Figure S7: FDTD-calculated and experimentally measured copolarized FF scattering patterns in the xz cross-section planes of the metasurface carpet cloak at different frequencies of 12.5~16 GHz in steps of 0.5 GHz under normal incidence of E→//y^. Figure S8: FDTD-calculated copolarized NF contour maps (top panel) and copolarized FF scattering patterns (bottom panel) in the xz plane of the large-angle metasurface cloak at different frequencies of 13.5 (left panel) and 14.5 GHz (right panel) under oblique incidence (θ = 30°) of (a) E→//x^, (b) E→//y^, (c) LCP, and (d) RCP wave. Figure S9: Experimental se [file 6382172.f1.doc]

Supplementary Materials

**Deterministic Approach to Achieve Full-Polarization Cloak**

By *He-Xiu Xu^1,2,6^*^*^*, Yanzhao Wang^1^, Chaohui Wang^1^, Mingzhao Wang^1^, Shaojie Wang^1^,* Fei Ding^3^, *Yongjun Huang^4^, Xiaokuan Zhang^1^, Haiwen Liu^5^*, *Xiaohui Ling^6^, Wei Huang*^2^*^*^*

*^1^Air and Missile Defense College, Air Force Engineering University, Xi'an 710051, China*

*^2^Institute of Flexible Electronics, Northwestern Polytechnical University, Xi'an 710072, China*

*^3^SDU Nano Optics, University of Southern Denmark, Campusvej 55, DK-5230 Odense, Denmark*

*^4^School of Information and Communication Engineering, University of Electronic Science and Technology of China, Chengdu 611731, China*

*^5^School of Electronic and Information Engineering, Xi’an Jiaotong University, Xi’an 710049, China*

*^6^College of Physics and Electronic Engineering, Hengyang Normal University, Hengyang 421002, China*

*Corresponding Authors: He-Xiu Xu, E-mail: hxxuellen@gmail.com; Wei Huang, E-mail: iamwhuang@nwpu.edu.cn

**1. Characterization of the meta-atom for** σ**-sensitive cloak**

The basic meta-atoms utilized to construct the σ-sensitive cloak is a reflective meta-atom which is composed of top Jerusalem-cross metallic pattern, an air spacer, and a backed metallic ground, as portrayed in Figure S1a. Here, the 4-mm-thick air layer is utilized to enhance the high-efficiency bandwidth and is not necessary if a tradeoff is considered between the mechanical strength and performance. The criterion of *r*_yy_ =*r*_xx_= 1 and *φ*_yy_ − *φ*_xx_ = 180° is utilized to guarantee the near-unity CP conversion efficiency by suppressing any component that does not carry PB phase information, as shown in Figure S1b. Here, we employ the dispersion engineering method to enable the slopes of *φ*_yy_ and *φ*_xx_ to be approximately equal within a broad bandwidth by modulating the length of three orthogonal bars of both “H” structures. As depicted in Figure S1c, after cautious optimizations, we precisely achieved *φ*_yy_ − *φ*_xx_ = 180° and near-unity CP cross conversion efficiency at 10.5 GHz. Such a high-efficiency CP reflection magnitude is necessary for the amplitude preservation of the cloak. Moreover, the CP cross-polarization conversion efficiency is more than 90% within 9.7~13.3 GHz, corresponding a fractional bandwidth of 34.2% with respect to the target frequency of 10.5 GHz. Figure S1d shows the theoretically calculated phase patterns of the half σ-sensitive metasurface cloak. Given this phase profile, the eventual cloak layout can be readily mapped out by spatially changing the orientation angles of each Jerusalem meta-atom according to the PB phase theory ($\delta\text{=}2\alpha$).


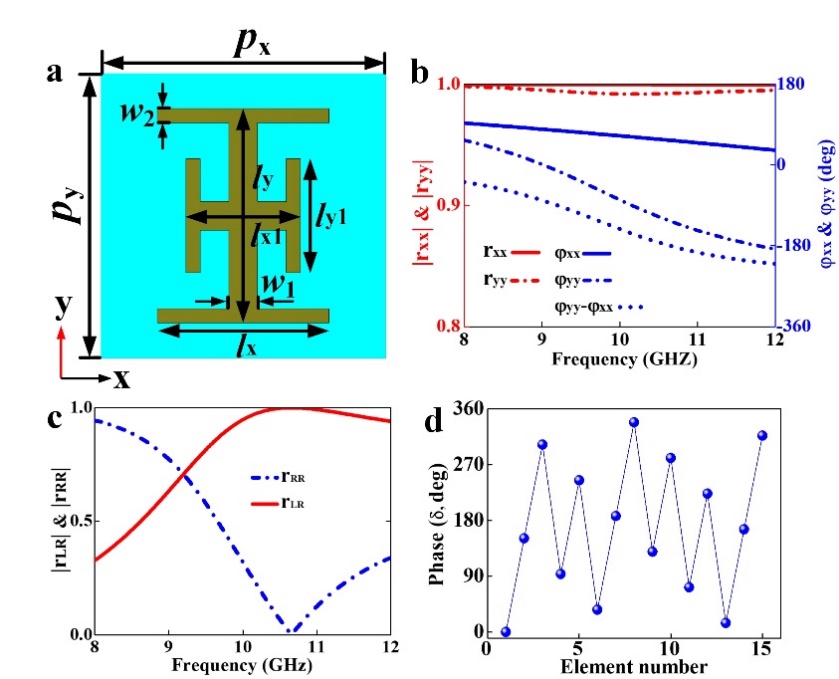


**Figure S1:** Topology and EM properties of the PB meta-atom utilized to construct the σ-sensitive metasurface cloak. (a) Meta-atom layout. EM response under normal illumination of (b) x/y-polarized LP wave and (c) CP wave. The geometrical parameters are optimized and detailed as *l*_x_ = 6, *l*_y_ = 7.5, *l*_x1_ = 5, *l*_y1_ = 4, *w*_1_ = 1, *w*_2_ = 0.5, and *h* = 4 mm.

**2. High-efficiency and broad bandwidth of the meta-atom**

In Figure 3 of the main text, we have shown the high-efficiency and 180^o^ phase control at two typical *β* and two representative frequencies. Here, we further afford the full amplitude and phase spectrum at all scanned frequencies and *β* under two cases of *θ*=0^o^ and *θ*=45^o^, see Figure S2. As is much appreciated, the near-unity high efficiency with continuous phase control by changing *β* from 10^o^ to 130^o^ is observed in both cases across a broad operation bandwidth. Such a level of broad bandwidth and high reflection rate is very beneficial for the design of frequency-insensitive cloak with preserved amplitude.

**
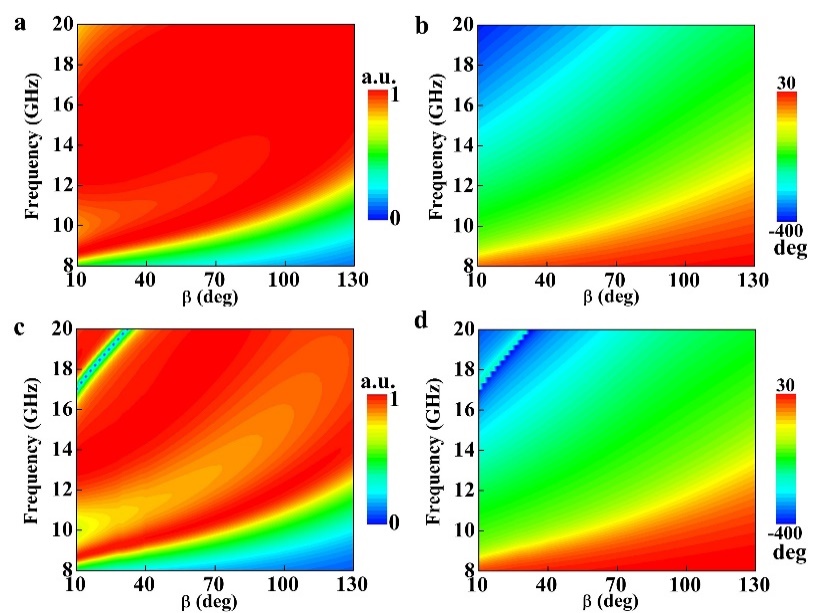
**

**Figure S2:** FDTD calculated cross-LP reflection (a, c) amplitude ($|r_{xy}|$) and (b, d) phase () of a basic meta-atom versus frequency and the parameter *β* under (a, b) normal (*θ*=0^o^) and (c, d) oblique (*θ*=45^o^) illumination.

**3. Bandwidth of the full-polarization metasurface illusion device**

In Figure 4 of the main text, we have discussed the invisible performance of 2D full-polarization illusion cloak at the center frequency of 14 GHz. Here, we further evaluate the illusion performance at other frequencies, aiming to illustrate the operation bandwidth. Figure S3 plots the FDTD calculated NF *E*_x_ distributions in xz cross-section plane at five representative σ states within 13~15 GHz. As is much appreciated, the tilted splitting V-shaped wavefront directing toward ±30^o^ is clearly observed in all cases and is quantitatively similar to that at center frequency 14 GHz in the main text. This proposal further finds strong support from the FF scattering patterns at other frequencies under $\overrightarrow{\text{E}}\text{//}\widehat{\text{x}}$, see Figure S4, where two highly-directive beams are clearly observed around ±30^o^. Therefore, the operation bandwidth is evaluated more than 2 GHz, corresponding to a fractional bandwidth of 14.3%. Slight distortion of fields at high frequency of 15 GHz is attributed to the small phase tolerances between the theoretical and physical values.


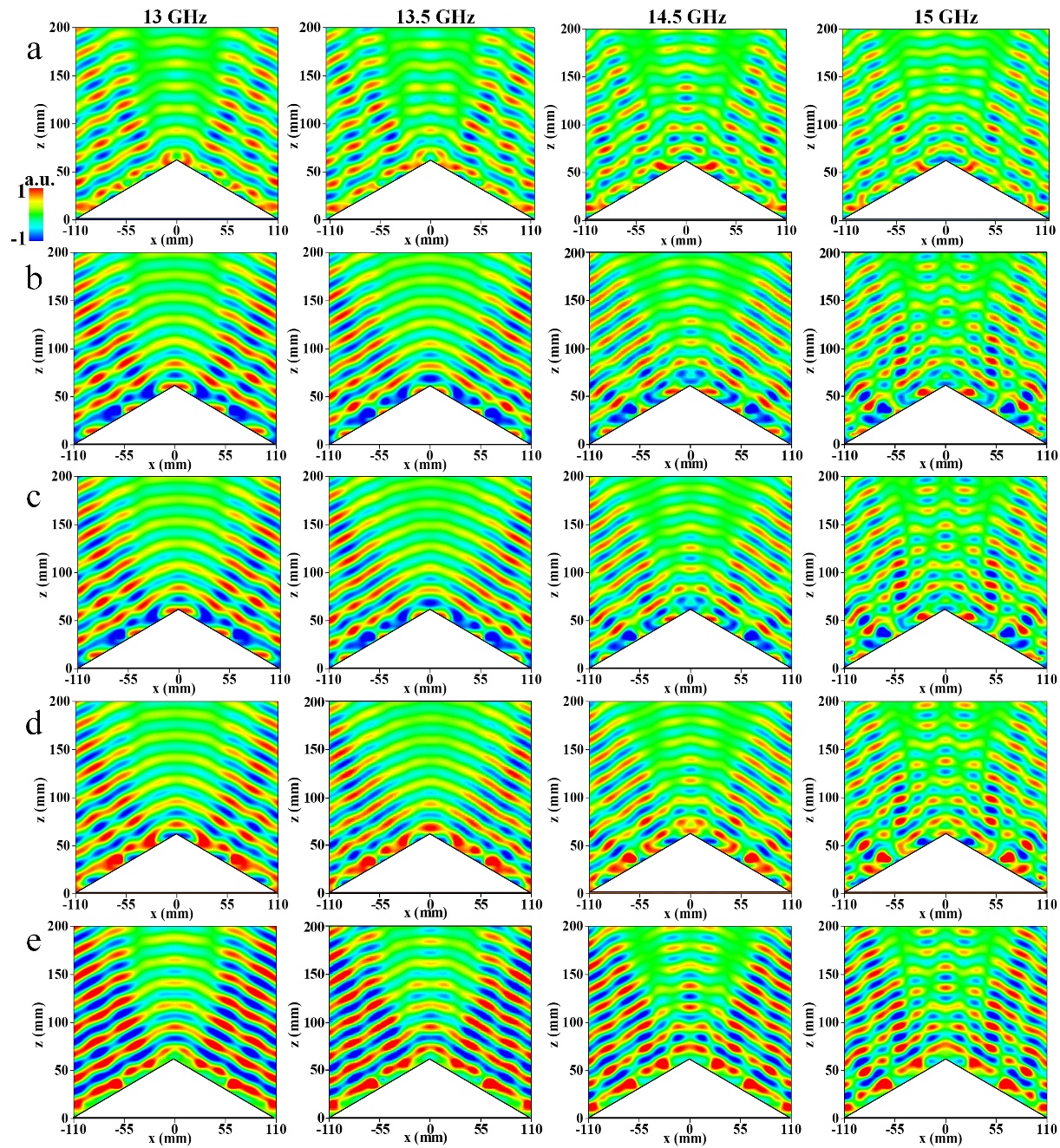


**Figure S3:** FDTD calculated co-polarized NF distributions in xz cross-section plane of the 2D metasurface illusion device at different frequencies of 13, 13.5, 14.5, and 15 GHz under normal incidence of $\overrightarrow{\text{E}}\text{//}\widehat{\text{x}}$, $\overrightarrow{\text{E}}\text{//}\widehat{\text{y}}$, $\overrightarrow{\text{E}}\text{//45}^{\text{o}}$, LCP and RCP wave.


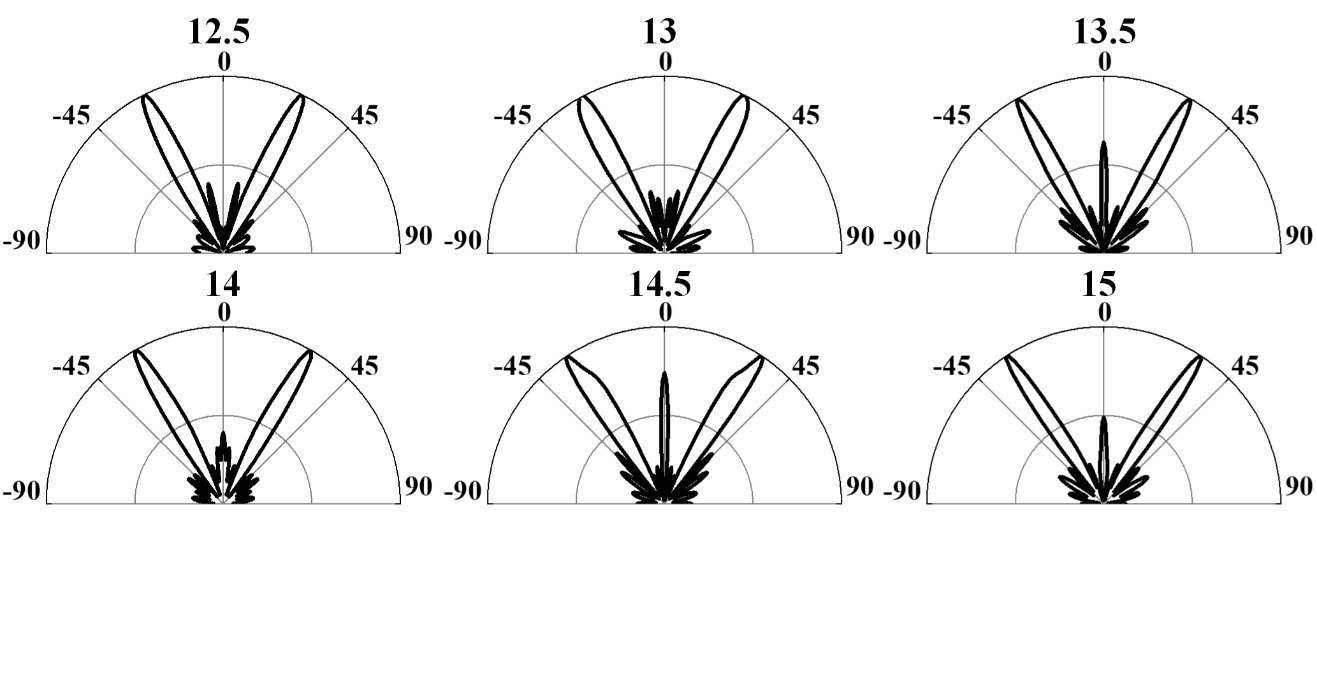


**Figure S4:** FDTD calculated co-polarized FF scattering patterns in xz cross-section planes of the 2D metasurface illusion device at different frequencies of 12.5~15 GHz in steps of 0.5 GHz under normal incidence of $\overrightarrow{\text{E}}\text{//}\widehat{\text{x}}$.

**4. Bandwidth of the full-polarization metasurface carpet cloak**

To further illustrate the working bandwidth of our metasurface carpet cloak, we also evaluate the NF and FF patterns in both xz and yz planes at several representative off-center frequencies within 13~16 GHz under five representative polarization states. Figures S5 and S6 display the FDTD and experimentally measured NF patterns, and Figure S7 compares the FF scattering patterns between FDTD calculations and experimental measurements. As shown in Figures S5 and S6, the V-shape wavefronts of the bare bump are flattened to great extent at all observed frequencies and polarization states except for slightly convex and concave wavefronts at edge frequencies. Nevertheless, such a level of distortion does not pose much penalty on the eventual cloaking performance. Such a proposal finds strong support from the FF scattering patterns depicted in Figure S7. Impressively, the single-mode highly-directive backward scattering is clearly observed at all observed frequencies within 13~15.5 GHz (an absolute 2.5 GHz bandwidth), corresponding to a fractional bandwidth of 17.9%. Such a level of bandwidth is very considerable relative to existing metasurface cloaks. Such mirror reflection illusion does not deteriorate until the working frequency moves to the low and upper edge frequencies of 12.5 and 16 GHz, where an amplitude null occurring at the broadside and the main beam is divided into two ones, which is obviously different from the mirror reflection of a flat metallic plate. Therein, the object will be detectable again from the background. The phase error induced by the fixed theoretical phase profile and the dispersive meta-atom give rise to the deteriorative off-frequency cloaking behavior.

**
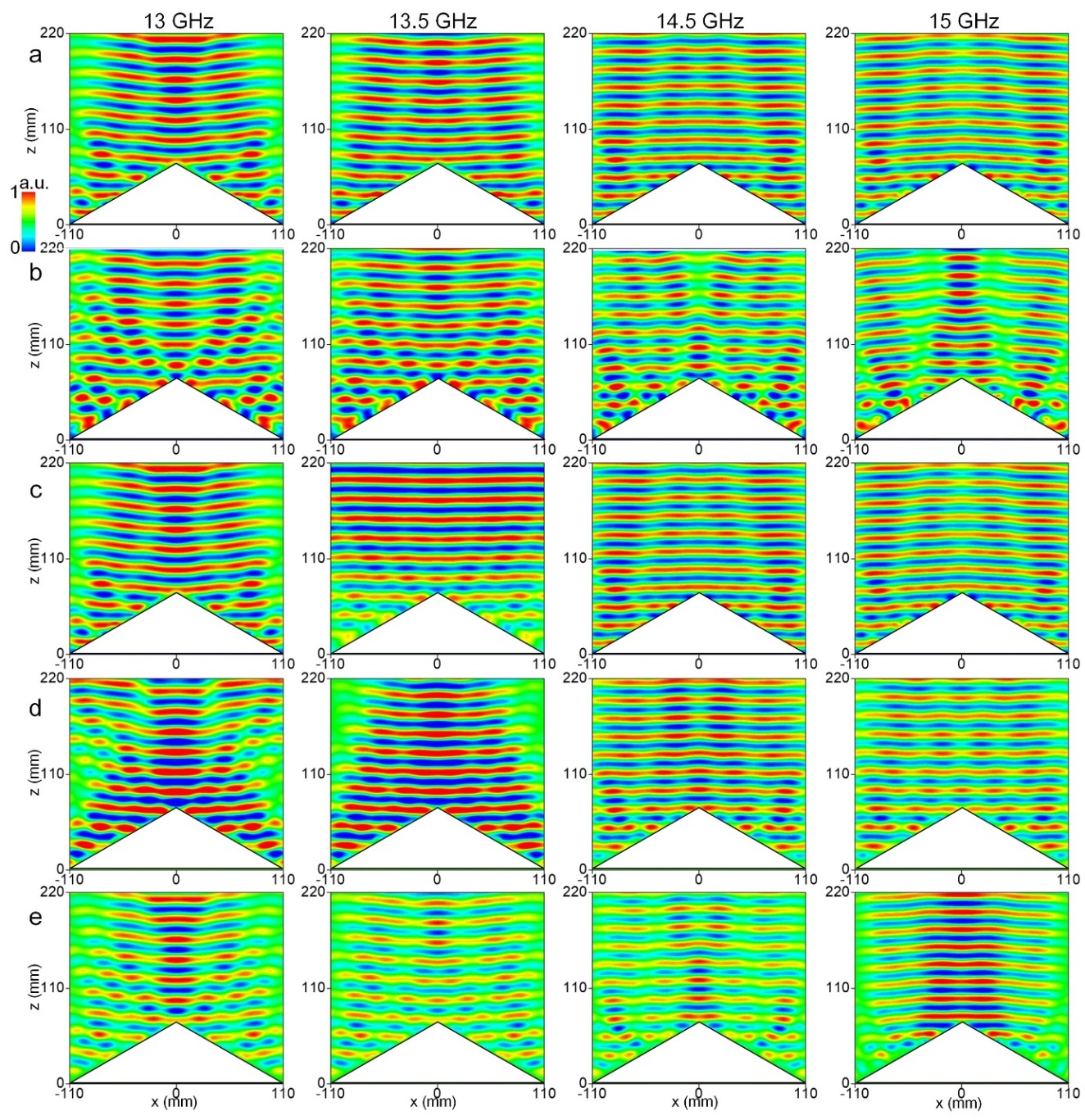
**

**Figure S5:** FDTD calculated co-polarized NF distributions in xz cross-section plane of the 2D metasurface carpet cloak at different frequencies of 13, 13.5, 14.5, and 15 GHz under normal incidence of $\overrightarrow{\text{E}}\text{//}\widehat{\text{x}}$, $\overrightarrow{\text{E}}\text{//}\widehat{\text{y}}$, $\overrightarrow{\text{E}}\text{//45}^{\text{o}}$, LCP and RCP wave.

**
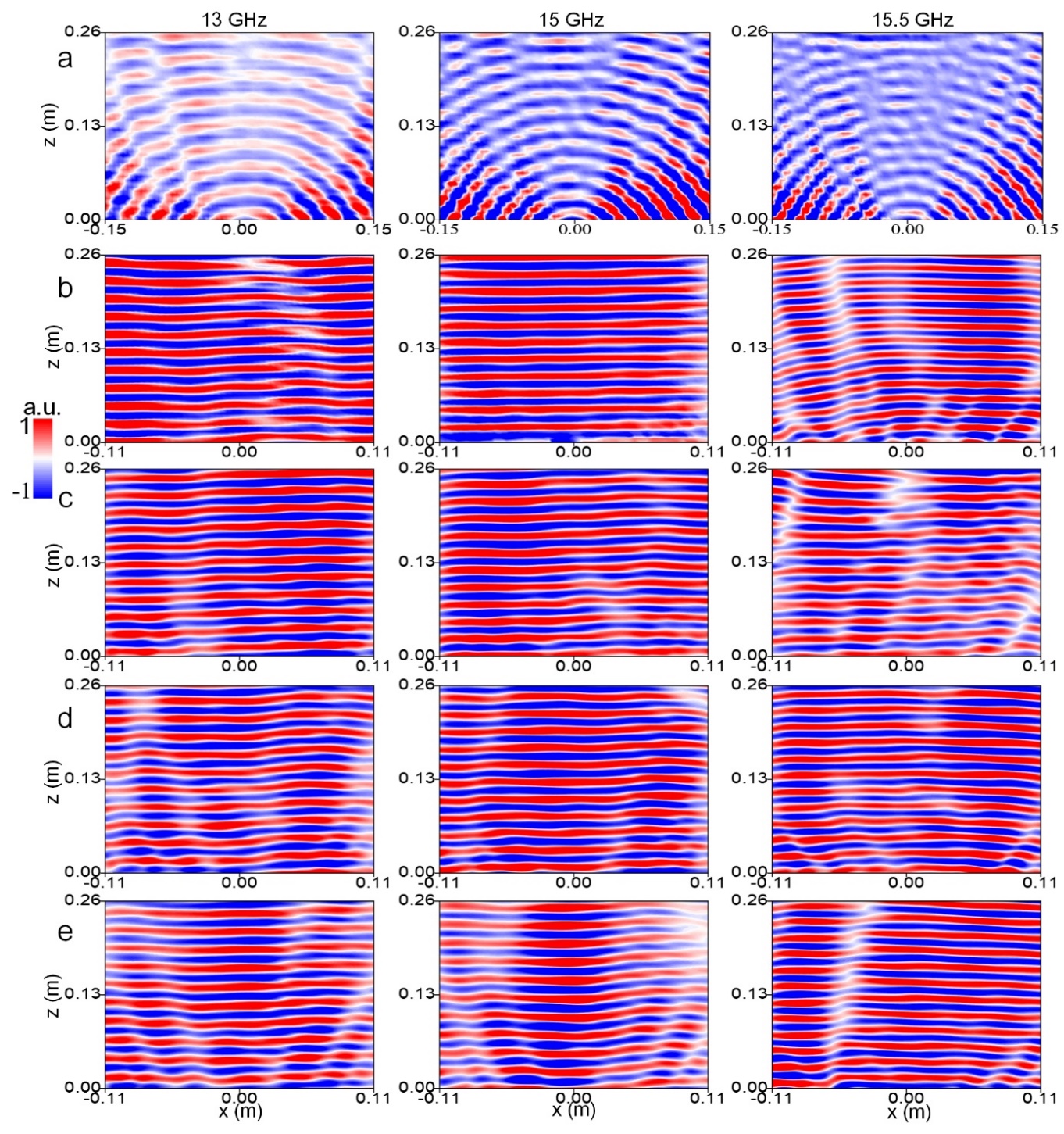
**

**Figure S6:** Experimentally measured NF distributions in xz cross-section plane of the (a) bare bump under $\overrightarrow{\text{E}}\text{//}\widehat{\text{y}}$ and metasurface carpet cloak at different frequencies of 13, 15 and 15.5 GHz under normal incidence of (b) $\overrightarrow{\text{E}}\text{//}\widehat{\text{x}}$, (c) $\overrightarrow{\text{E}}\text{//}\widehat{\text{y}}$, (d) LCP and (e) RCP wave. The *E*_y_ maps are plotted in $\overrightarrow{\text{E}}\text{//}\widehat{\text{y}}$, LCP and RCP cases while *E*_x_ maps are in $\overrightarrow{\text{E}}\text{//}\widehat{\text{x}}$ case.


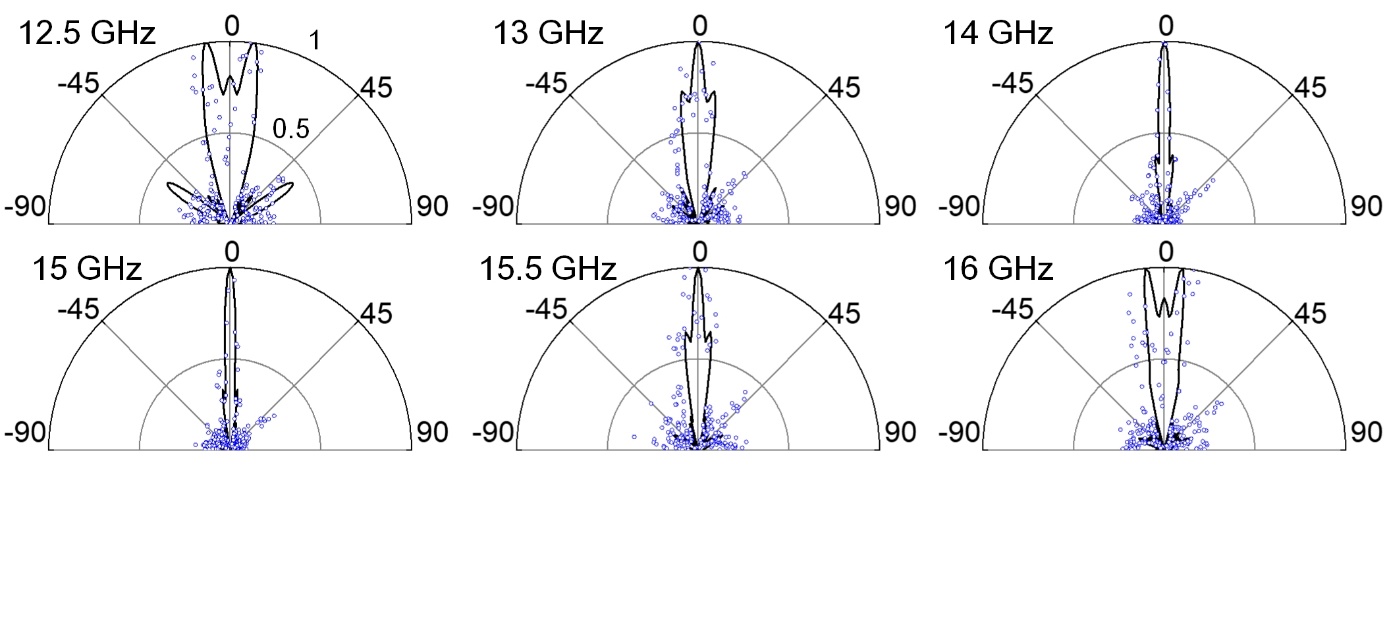


**Figure S7:** FDTD calculated and experimentally measured co-polarized FF scattering patterns in xz cross-section planes of the metasurface carpet cloak at different frequencies of 12.5~16 GHz in steps of 0.5 GHz under normal incidence with the incident polarization of $\overrightarrow{\text{E}}\text{//}\widehat{\text{y}}$.

**5. Bandwidth of the full-polarization large-angle metasurface cloak**

To illustrate the bandwidth of large-angle metasurface cloak, Figure S8 shows the FDTD calculated NF and FF scattering patterns under obliquely incident x-polarized EM wave with *θ*=30^o^ at two representative frequencies of 13.5 and 14.5 GHz. As expected, both NF and FF results reveal a tilted wavefront and beam toward the angle of *θ*=-30^o^. Slight fluctuations of fields are inspected across specific positions at specific frequencies due to the non-uniform reflection amplitude of spatially varied meta-atoms at oblique incidence, as discussed in the main text. This is especially true for the non-uniform amplitudes of meta-atoms at two slopes induced by much deviated incidence angle (*θ*=90^o^ and 0^o^ for meta-atoms at left and right slopes) at oblique incidence. Such asymmetry non-uniform amplitudes of meta-atoms lead to sharply varied cloaking performance (deteriorative wavefront and large sidelobes) as frequency deviates from the center one, resulting in a narrower operation bandwidth (1 GHz) with respect to that at normal incidence.


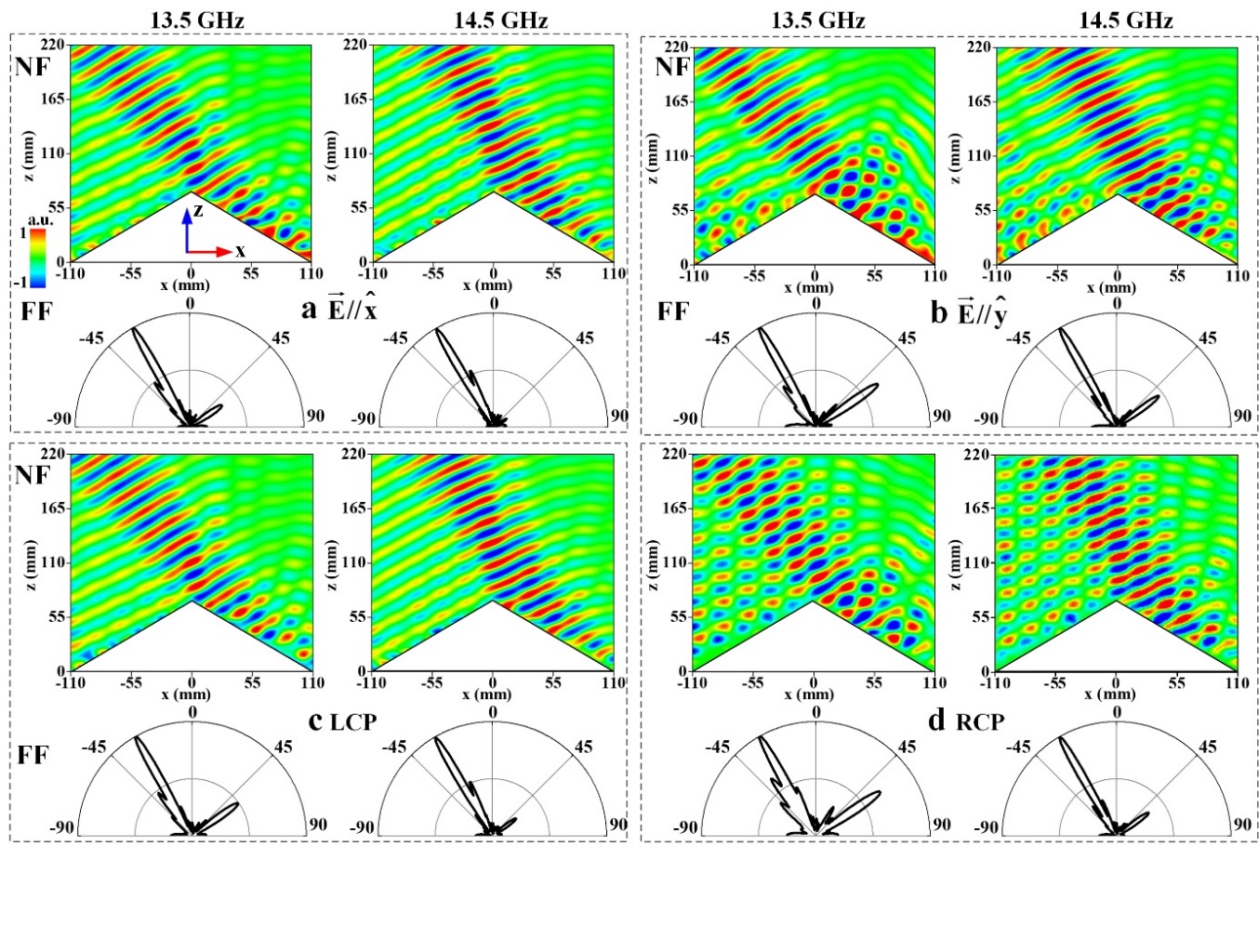


**Figure S8:** FDTD calculated co-polarized NF contour maps (top panel) and co-polarized FF scattering patterns (bottom panel) in xz plane of the large-angle metasurface cloak at different frequencies of 13.5 (left panel) and 14.5 GHz (right panel) under oblique incidence (*θ*=30^o^) of (a) $\overrightarrow{\text{E}}\text{//}\widehat{\text{x}}$, (b) $\overrightarrow{\text{E}}\text{//}\widehat{\text{y}}$, (c) LCP and (d) RCP wave.

**6. NF and FF experimental setup**


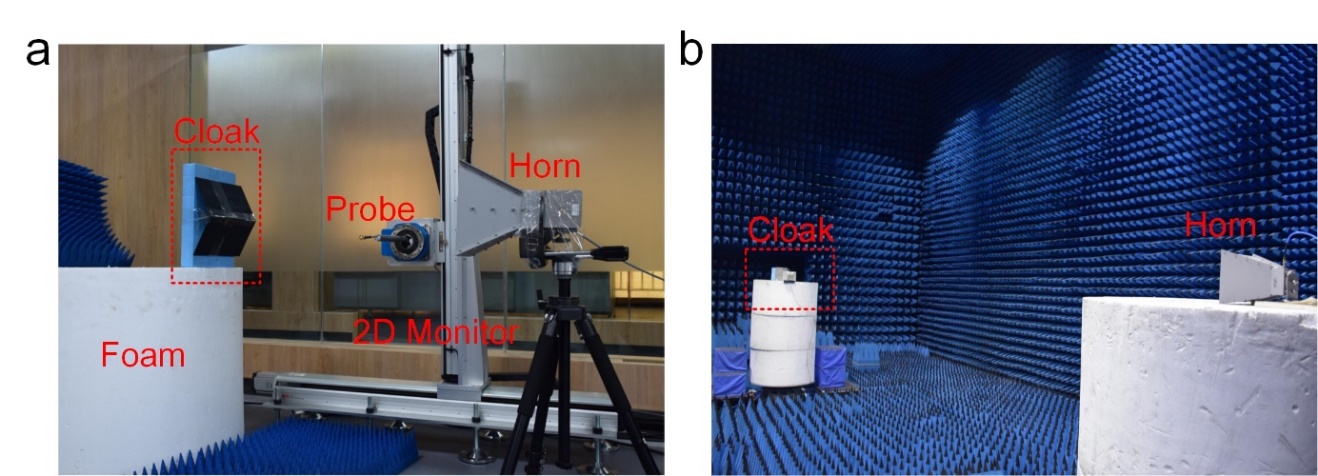


**Figure S9:** Experimental setup for (a) NF and (b) FF measurements.
